# 

**DOI:** 10.1192/bjb.2025.21

**Published:** 2026-02

**Authors:** Lindsey McKeown

**Affiliations:** NHS Scotland, Glasgow, UK.

**Figure:**
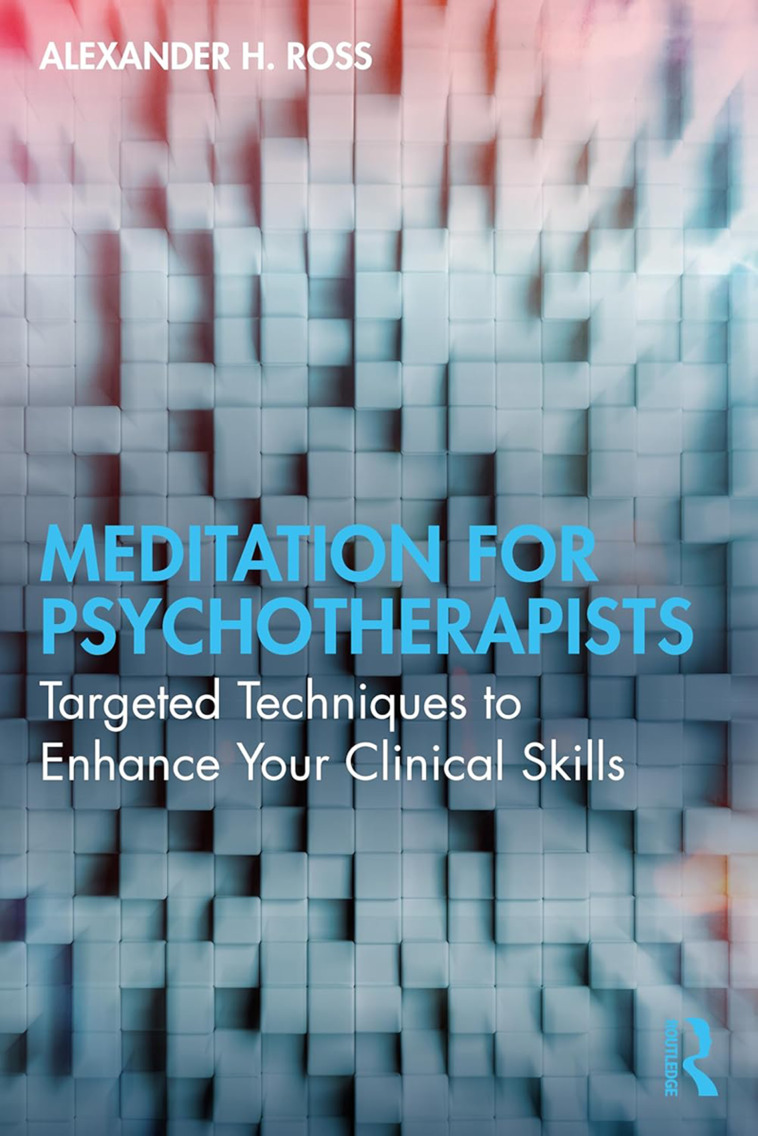

This book is a thought-provoking and genuinely interesting exploration of how meditative practices can be integrated into psychotherapeutic practice. The author encourages the reader to approach the subject with an open mind, while recognising that themes within the book may seem abstract or even alienating to those with a strong scientific grounding.

Ross succinctly explains that these meditative exercises are being suggested as an adjunct to traditional psychotherapeutic training rather than a replacement. We are gently led through a range of meditation exercises that align with specific therapeutic mindsets (including psychoanalysis, person-centred therapy and mentalisation-based therapy), and reminded that these practices are designed to be done outside of therapy sessions, to develop a therapeutic stance.

Certain aspects of the book can be fairly dense, but are never overwhelming, and Ross expertly tackles some fairly weighty concepts with ease and, quite enjoyably, humour! He clearly introduces the fundamentals of meditation, while helpfully providing end-of-chapter summaries to consolidate the content. Interestingly, the discussion becomes even more illuminating when Ross defines meditation by what it is *not* (p. 11).

Ross is able to consistently ground his meditative exploration in psychoanalytic theory and neuroscientific perspectives, which provides a rich (and often quite fascinating) context to the explanations. He is also particularly skilled in pinpointing the essence of being a therapist: ‘the therapist is in the midst of an emotional experience with the client so must be sensitive albeit robust, focussed but also receptive and maintaining an empathetic and resilient presence’ (p. 23). Even if the reader struggles to connect with the meditative aspects of the book, we are consistently grounded in the reality of what it means to be a compassionate and thoughtful clinician. What really stands out is the author’s humility and vulnerability as he sprinkles in his own personal experiences of meditation throughout the text.

*Meditation for Psychotherapists* is perhaps not a book to be consumed in one sitting; rather, it benefits and even thrives from careful reflection and the re-reading of certain chapters. It is a refreshing read.

